# Structure, Evolution, and Functions of Bacterial Type III Toxin-Antitoxin Systems

**DOI:** 10.3390/toxins8100282

**Published:** 2016-09-28

**Authors:** Nathalie Goeders, Ray Chai, Bihe Chen, Andrew Day, George P. C. Salmond

**Affiliations:** Department of Biochemistry, University of Cambridge, Cambridge CB2 1QW, UK; ng394@cam.ac.uk (N.G.); rc636@cam.ac.uk (R.C.); bc407@cam.ac.uk (B.C.); awd33@cam.ac.uk (A.D.)

**Keywords:** abortive infection, altruistic suicide, type III toxin-antitoxin, bacteriophages, quaternary structures, co-evolution, pseudoknotted RNA, endoribonuclease

## Abstract

Toxin-antitoxin (TA) systems are small genetic modules that encode a toxin (that targets an essential cellular process) and an antitoxin that neutralises or suppresses the deleterious effect of the toxin. Based on the molecular nature of the toxin and antitoxin components, TA systems are categorised into different types. Type III TA systems, the focus of this review, are composed of a toxic endoribonuclease neutralised by a non-coding RNA antitoxin in a pseudoknotted configuration. Bioinformatic analysis shows that the Type III systems can be classified into subtypes. These TA systems were originally discovered through a phage resistance phenotype arising due to a process akin to an altruistic suicide; the phenomenon of abortive infection. Some Type III TA systems are bifunctional and can stabilise plasmids during vegetative growth and sporulation. Features particular to Type III systems are explored here, emphasising some of the characteristics of the RNA antitoxin and how these may affect the co-evolutionary relationship between toxins and cognate antitoxins in their quaternary structures. Finally, an updated analysis of the distribution and diversity of these systems are presented and discussed.

## 1. Introduction

Toxin-antitoxin (TA) systems are composed of a bacteriostatic or bactericidal toxin and a cognate antidote which is referred to as the antitoxin. In most cases, the antitoxin directly interacts with either the proteinaceous toxin or its mRNA and thus antagonises the deleterious effect of the toxin. In addition to their physical interdependence, they are linked at the genetic level and are often encoded in bicistronic operons with a promoter-proximal antitoxin gene. Save for a few Type I systems, all TA systems are encoded in operons wherein the antitoxin gene is usually found upstream of the toxin-encoding ORF. Nonetheless a few Type II systems such as HigBA and HicAB are exceptions and have a reverse genetic organisation [1]. This transcriptional organisation favours an excess of antitoxin in homeostatic conditions where the toxin is inhibited. The harmful activities of the toxins are due to their interference with essential cellular processes including DNA replication, translation, cell wall synthesis, and maintenance of membrane integrity [2,3,4,5,6].

The active toxin always interacts with its targets as a protein while the nature of the potent antitoxin is either RNA or protein. In addition, antitoxins neutralise their cognate toxins at several levels and act via distinct mechanisms. The nature of the antitoxin and its mode of action underpin the classification of TA systems into five Types (I–V) [7,8,9]. RNA antitoxins of Type I and III interact with the toxin transcripts (RNA-RNA interactions) or with the toxic protein (RNA-protein interactions) respectively. Most Type I antitoxin RNAs bind the toxin transcript in its 5'UTR region. Formation of this RNA-RNA duplex has two main effects [10]. Firstly, translation of the mRNA into the toxic protein is hindered as the antitoxin RNA is usually complementary to the region containing the ribosome binding site (RBS) of the toxin transcript or directly competes with ribosomes. In addition to blocking translation initiation, the antitoxin/toxin RNA duplexes are the targets of cellular RNases and thus antitoxin binding ultimately leads to degradation of the toxin transcripts [6]. Aside from Type I TA systems, inhibition of toxin translation is also used by the only Type V system identified so far [7]. In this case, toxin production is turned off directly by the antitoxin which is an RNase that degrades toxin mRNAs and thus directly regulates toxin transcript levels [7]. In Type II, III, and IV systems, translation of the toxin is not directly affected. Type II systems use direct protein-protein interactions where antitoxins either mask the toxin active site or sterically hinder the toxins from reaching their target [1,2,3]. Type IV antitoxins also prevent toxin-target interactions but achieve this by competing with the toxins for their target, without direct contacts with their cognate toxins [8,9]. Finally, Type III TA systems fall into the category of RNA-protein interactions in which the toxin active site is occluded by the antitoxin RNA [11,12,13,14].

TA systems were originally discovered in the late 80s [15,16] and, for a long period, were defined in only two types (Type I and II) which have been extensively studied. In contrast, the archetypal Type III TA system and, subsequently, the Type IV and V systems, were discovered only recently. While data are still comparatively limited for the newer Type IV and V systems, a more holistic image is beginning to emerge regarding Type III systems, with accumulating biochemical, structural, and functional data. This review covers these systems, describing their diversity and toxin-antitoxin/abortive infection bifunctionality and discusses their impact on bacteria-phage co-evolution, given their anti-phage activity.

## 2. Type III TA Systems Are Split into Three Families Which Share the Same Genetic Organisation

The novelty of Type III TA systems involves the nature of RNA-protein interactions between their components [11,12]. These interactions are unusual as the toxin is involved in processing the antitoxin into its active form. More precisely, the antitoxin, a small non-coding RNA (sRNA) composed of several repeats of short nucleotide sequences, is processed into monomers of these repeats by the toxin. The heteromeric complexes adopted by Type III systems during homeostatic conditions are composed of alternating interactions between antitoxin and toxin monomers [12,13,14]. As for other Types of TA systems, the antitoxin has a shorter half-life than the toxin [11] but the specific details of antitoxin degradation are not yet completely clear.

**Figure 1:**
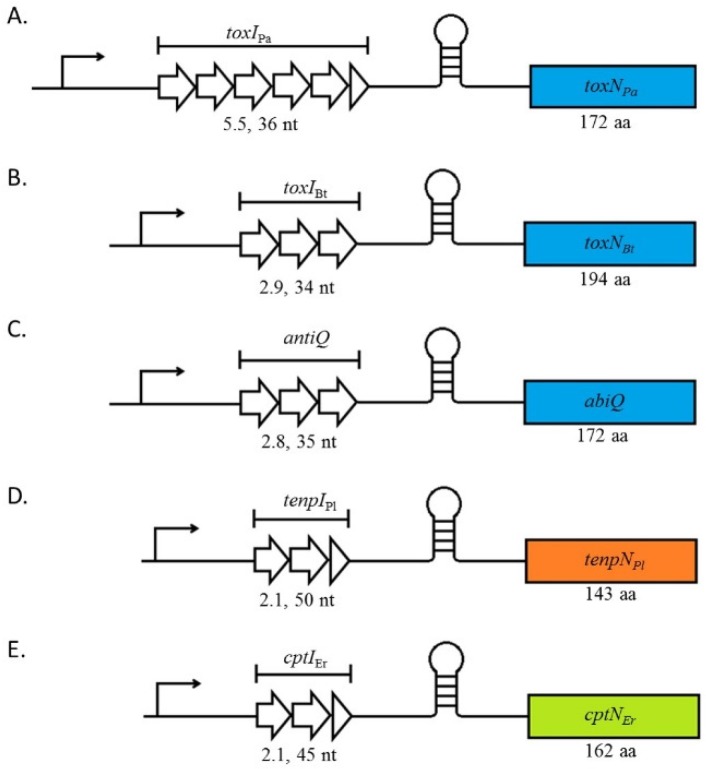
Genetic organisation of Type III systems. Type III systems are arranged with the antitoxin gene preceding that of the toxin—separated by a Rho-independent terminator. Five Type III TA loci are shown: (**A**) *toxIN_Pa_* located on pECA1039 from *Pectobacterium atrosepticum*; (**B**) *toxIN_Bt_* located on pAW63 from *Bacillus thuringiensis*; (**C**) AbiQ located on pSRQ900 from *Lactococcus lactis*; (**D**) *tenpIN_Pl_* from the chromosome of *Photorhabdus luminescens*; and finally (**E**) *cptIN_Er_* from the chromosome of *Eubacterium rectale*.

At the genetic level, Type III TA systems are organised in characteristic bicistronic operons transcribed from a single promoter. The downstream toxin gene is preceded by a Rho-independent terminator that separates it from the upstream short repetitive nucleotide sequences that encode the antitoxin sRNA (Figure 1) [11,17]. Presumably, organisation in operons ensures a higher synthesis of the antitoxin compared to the toxin and thus avoids physiologically precocious, and potentially lethal, toxin activity. Unique to Type III systems toxin expression is further modulated by the presence of the inter-gene Rho-independent terminator. A final regulatory fail-safe may reside in the fact that one antitoxin sRNA will be processed into several monomers that could neutralise two or three toxins, thus further ensuring an appropriately regulated antitoxin:toxin stoichiometry.

Although all Type III TA systems share the same genetic arrangement, they can be further differentiated into three families which are classified according to the amino acid sequence similarities that they share [18]. The subfamilies are called ToxIN, CptIN, and TenpIN where the “I” and “N” represent the antitoxin and toxin components respectively. Thus, for the ToxIN system of *Pectobacterium atrosepticum* the antitoxin is referred to as ToxI*_Pa_*, the toxin as ToxN*_Pa_* and both components as ToxIN*_Pa_* [18]. CptIN was named after the *Coprococcus catus* GD/7 system (*CoPrococcus* Type III Inhibitor/toxiN) and the third family, TenpIN for Type III ENdogenous to *Photorhabdus* Inhibitor/toxIN [18]. While the toxin sequence directly influences the subgroup to which a particular system belongs, it is also interesting to note how their cognate antitoxins differ between and within the subgroups.

## 3. Antitoxin Length Is Important for Type III System Functions

Antitoxin repeats are a key feature of Type III systems. The number of repeats varies between systems and they have been shown to be crucial for antitoxin activity. For instance, the antitoxins of the ToxIN*_Pa_*, ToxIN*_Bt_*, and AbiQ systems that belong to the ToxIN family, all diverge at the primary sequence level and number of repeats, while the length of the monomers is quite conserved. ToxI*_Pa_*, ToxI*_Bt_*, and *antiQ* sRNAs are composed of, respectively, 36 nucleotides repeated 5.5 times, 34 nucleotides repeated 2.9 times, and 35 nucleotides repeated 2.8 times (Figure 1). In vitro, the antitoxin activity can be retained despite increasing or decreasing repeat numbers. However, the range of repeats in which each antitoxin remains functional varies. For instance, 2.5 repeats from 5.5 were necessary and sufficient for ToxI*_Pa_* antitoxin to inhibit its toxin [19] while at least 1.8 repeats from 2.8 were essential for the antitoxin activity of *antiQ* [17]. *antiQ* mutants containing 1.8 and 3.8 repeats were readily obtained while clones with only 0.8 of a basic repeat were inviable, suggesting that an incomplete repeat sequence is insufficient to avoid toxicity of AbiQ [17]. In addition to its TA function, the AbiQ system also acts as an abortive infection system against some phages (See below, Section 6.1). This activity is also affected by the number of *antiQ* repeats however the anti-phage activity of the system is altered independently of its toxin neutralising activity. For instance, deletion or addition of one repeat to *antiQ* decreased the phage resistance provided by the AbiQ system, indicating that the length of the wild-type *antiQ* is critical for optimal anti-phage activity. Similarly, mutations in key residues for antitoxin processing led to significant loss of anti-phage activity while a point mutation that affects pseudoknot structure increased anti-phage activity, but did not affect bacterial fitness [17].

## 4. Assembly of the Toxin-Antitoxin Complexes

When the paradigmatic ToxIN*_Pa_* system was first discovered, the activity of the toxin component was unknown and mining structural databases with the predicted structure of ToxN*_Pa_* gave no meaningful results [11]. Insight into its activity was gained later with the resolution of its crystal structure and the discovery of the triangular architecture adopted by the three toxin-antitoxin monomers [12]. Resolution of the quaternary structures of further Type III systems showed that this interesting feature of Type III TAs exhibits some variations on a theme where toxin and antitoxin monomers alternate (in hexameric or tetrameric complexes) in which only RNA-protein interactions occur. A hallmark shared by all the structures is that it is the antitoxin processing that leads to the inactive, stable TA complex [12,13,14]. So far, the core architecture of Type III systems seems to be subfamily specific and likely depends on the length and fold of the antitoxin monomers.

### 4.1. The ToxIN Systems Form Triangular Heterohexamers

**Figure 2:**
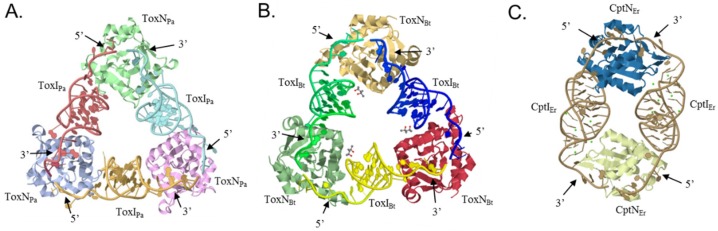
Crystal structures of Type III TA systems. (**A**) ToxIN*_Pa_* (PDB ID: 2XDD) and (**B**) ToxIN*_Bt_* (PDB ID: 4ATO) form heterohexameric complexes [12,13]; (**C**) CptIN*_Er_* (PDB ID: 4RMO) assembles into a heterotetrameric complex [14].

Most of the structural data currently available concerns the ToxIN subfamily. The quaternary structure of the ToxIN*_Pa_* and ToxIN*_Bt_* systems has been resolved (Figure 2A,B) and bioinformatic analyses predict that the AbiQ system shares the same quaternary architecture [12,13,20]. These crystal structures provided important insights into the mechanism of RNA anti-toxicity.

Both the ToxIN*_Pa_* and ToxIN*_Bt_* systems assemble into heterohexameric complexes (Figure 2A,B) that adopt a triangular architecture where toxins occupy the apices and are held together by processed antitoxin monomers [12,13]. In the complexes, each pseudoknotted ToxI RNA is bound head to tail to two separate ToxN monomers and occludes their active sites. Since ToxN*_Pa_* and ToxN*_Bt_* are both endoribonucleases, cleavage of cognate tandem RNAs into single repeats and assembly into the ToxIN complexes is likely associated with the inhibition of toxicity. Experimental data show that, while ToxN*_Pa_* is neutralized by both the processed and precursor forms of ToxI*_Pa_*, the precursor ToxI*_Pa_* is the preferred substrate, indicating the involvement of the antitoxin processing in toxin inhibition [13]. Additionally, the pseudocontinuous arrangement of ToxI units in these structures and the 2′-3′ cyclic phosphates at the 3′ end of the processed antitoxins support this mechanism [12,13]. Finally, ToxIN*_Pa_* complexes can self-assemble in vitro from ToxN*_Pa_* combined either with processed or precursor ToxI*_Pa_* RNAs indicating that the self-assembly of these structures is largely mediated by the antitoxin RNAs and does not require any cellular factors or exogenous energy [13].

Both ToxI*_Pa_* and ToxI*_Bt_* antitoxin monomers fold into a classic H-type pseudoknot structure flanked by two single-stranded tails. These ends and the adjacent areas of the pseudoknot interact with their respective toxins to stabilise the trimeric structure (Figure 2A,B) [12,13]. Analysis of the crystal structure of ToxIN*_Pa_* showed that each ToxI*_Pa_* tail interacts with a different ToxN*_Pa_* monomer via electropositive grooves where hydrogen bonds occur between the protein and the RNA bases. The antitoxin 3′ end tail containing the 2′-3′ cyclic phosphate is held in place in this groove by five side chains, Tyr29, Lys33, Thr52, Ser53, and Lys55, that form the ToxN*_Pa_* active site [12]. Similarly, key areas of ToxI*_Bt_* that interact with ToxN*_Bt_* are the single stranded tails, where C19 and G20 interact with Lys148 of the toxin, and the 5′ tail of ToxI*_Bt_* and U10 interact with a hydrophobic pocket of the ToxN*_Bt_* interacting with the 3′ end [13].

### 4.2. CptIN_Er_ Assembles into Heterotetramers

In contrast to the ToxIN systems, CptIN*_Er_*—currently the only other Type III TA system with a solved crystal structure—assembles into tetramers (Figure 2C). Its quaternary structure is composed of two toxin monomers joined by two antitoxin RNAs [14]. The CptI monomers are longer than the corresponding examples in the ToxI repeats and the fold they adopt probably accounts for the difference in quaternary structures—with the nature of the pseudoknotted RNA perhaps driving the evolution of the toxin-antitoxin complexes.

### 4.3. Type III Antitoxins Form Pseudoknots

**Figure 3:**
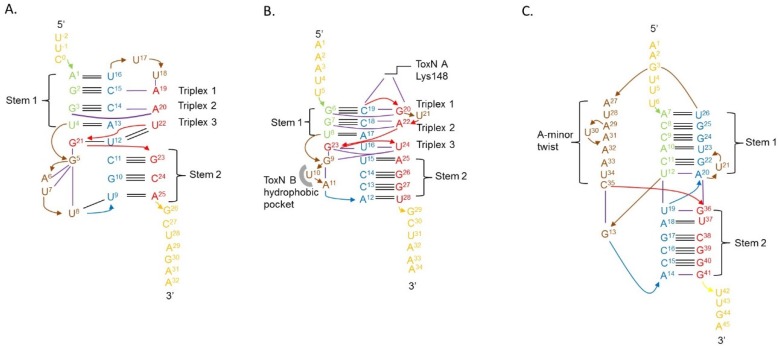
Pseudoknot arrangements of Type III antitoxins. (**A**) ToxI*_Pa_*; (**B**) ToxI*_Bt_*; and (**C**) CptI*_Er_*. Base-base hydrogen bonds are shown by black lines. Nucleotides involved in loops are indicated in brown. Corresponding areas for each antitoxin are highlighted by similar colors.

All the antitoxins crystallised so far adopt a pseudoknotted fold in their TA complexes. Pseudoknots are a recurrent RNA structural motif in which a loop forms interactions with distal bases outside the loop to form triple stranded structures. Usually the third nucleotide is an adenine enabling A-minor interactions [21]. In the quaternary structures of the TA systems, the antitoxin pseudoknots exhibit three distinct regions that interact with each other through duplex and triplex hydrogen bonds (Figure 3) [12,13]. An important feature is the core of the pseudoknot which contains three internal base triplexes. One of these internal base triplexes (triplex 3, GUU) separates the two based-paired stems of the pseudoknot with interdigitation of a guanine (Figure 3A,B). While key components are conserved in both ToxI pseudoknots, this curious structural aspect was not predicted due to the low sequence homology between the antitoxins. Despite overall similar structures, the RNA-protein interfaces show substantial differences as highlighted by the selective inhibition displayed by both antitoxins and the absence of functional cross-talk between antitoxins and toxins of the *Pectobacterium* and *Bacillus* systems [13].

In contrast, CptI*_Er_* monomers fold into an H-type pseudoknot distinctly different from the fold of the antitoxins of the ToxIN family. Firstly, CptI*_Er_* has two coaxial stems assembled entirely from duplex base pairing (Figure 3C) as opposed to the triplex base pairing seen in the other two antitoxins [14]. Even among RNA pseudoknots, the loops of the CptI*_Er_* pseudoknots are unusual. Indeed, loop 1 (L1) interacts with stem 2 (S2) while loop 2 (L2) interacts with stem 1 (S1), a configuration that leads often to triplex base pairing [21]. These canonical pseudoknot interactions are found in the ToxI RNAs where two triplex base pairs occur from the interaction of L2 with S1, although there is no triplex pairing with L1 and S2 (Figure 3A,B) [12,13]. The CptI*_Er_* pseudoknot is more atypical as L1 is extremely short and only consists of a single base that does not interact with S2 and is instead held in place by interactions with the 3′ end of L2 (Figure 3C) [14]. L2 of CptI*_Er_* is adenine rich and longer than in the other antitoxins and forms a novel counter-clockwise A-minor twist motif [14]. This conformation appears to be necessary for its antitoxin activity, as suggested by experiments on substitution and deletion mutations that disrupted key features of this twist [14]. As with the ToxI-type antitoxins, functional cross inhibition experiments have confirmed that CptI antitoxins also show high toxin specificity [14].

## 5. Type III Toxins Share a Common Fold and Activity

To date, the structures of four Type III toxins have been solved; three of which belong to members of the ToxIN family (ToxIN*_Pa_*, ToxIN*_Bt_* and AbiQ) and, more recently, a member of the CptIN family. All toxins share a globular β core surrounded by α-helices and loops. While the core structure is conserved, most variations are found on the surface. These variations are thought to account for cleavage and antitoxin specificity of the toxins. All Type III toxins tested so far for their mechanistic action have been shown to be endoRNases that cleave mRNAs in adenine-rich regions but with slightly different substrate specificities: ToxN*_Pa_* AA/AU, ToxN*_Bt_* A/AAAA, and AbiQ A/AAA [13,20]. As for other toxins that inhibit translation, Type III toxins initially have a bacteriostatic effect on growth [3,11,20] that ultimately leads to lethality.

### 5.1. The ToxN Family

**Figure 4:**
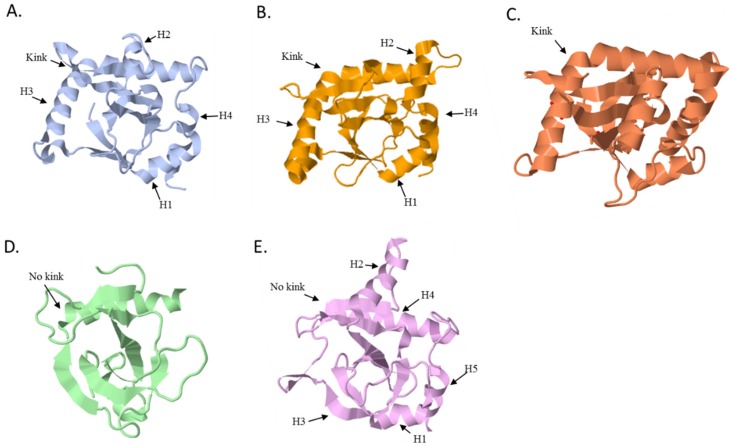
Structures of the toxins. (**A**) Structure of ToxN*_Pa_* (PDB ID: 2XDD, [12]); (**B**) ToxN*_Bt_* (PDB ID: 4ATO, [13]); (**C**) AbiQ (PDB ID: 4GLK, [20]); (**D**) Kid (PDB ID: 1M1F, [22]); and (**E**) CptN*_Er_* (PDB ID: 4RMO, [14]). The presence or absence of the kink in helix 3 (H3) is indicated for each toxin.

ToxN*_Pa_* and ToxN*_Bt_* have the same highly twisted core of six anti-parallel β-sheets and five main variable loop regions (Figure 4A,B) [12,13]. Four of these loops, especially the long kinked helix H3, act as the major sites of interaction between the toxins and their respective antitoxins while the fifth encompasses the active site. In line with the structural data, bioinformatics analysis of the sequences of the other members of the ToxN family showed that the core fold is conserved while most sequence variability clusters in regions corresponding to these five loops [12]. The structure of AbiQ also encompasses a core of six-stranded anti-parallel β-sheets surrounded by 6 α-helices (Figure 4C) [20]. Surprisingly, the RNase activity of AbiQ was only eliminated by one site-directed mutation in Ser51Leu which is thought to make the nucleophilic attack during the cleavage [20]. The RNase activity of AbiQ was retained when replacing the Ser51 by a threonine, the equivalent residue found in the active site of ToxN [12,13,20].

### 5.2. The CptN Family

CptN*_Er_* shares the same core fold found in the other toxins despite its lower primary sequence identity with members of the ToxN family (Figure 4E). In CptN*_Er_*, the highly twisted anti-parallel β-sheets that form the core are surrounded by four α-helices that make extensive interactions with the cognate antitoxin sRNA [14]. Compared to the ToxN family, in which ToxN*_Pa_*, ToxN*_Bt_*, and AbiQ have a kink in helix three [12,13,20] the equivalent helix in CptN*_Er_*, helix H4, is much shorter [14]. However many of the hydrogen bonding networks in the RNase active site are also conserved suggesting that this toxin is also an endoRNase.

### 5.3. Type III Toxins Share Homology with Type II Toxins

Unexpectedly, the structure of Type III toxins shows significant homology with toxins from the Type II MazF/Kid/CcdB family (Figure 4). Members of this Type II family share a similar fold despite low sequence similarity and different mechanisms of action. For instance, Kid and MazF toxins inhibit translation by acting as endoRNases while CcdB toxins interact with DNA gyrase and affect DNA replication [23,24,25]. Type II and Type III toxins share very low sequence similarity, e.g., only 11% identity between ToxN*_Pa_* and Kid, but their respective structures encompass a similar β core fold region surrounded by helices and loops. Interestingly, despite sharing the same overall structure and the same molecular activity i.e., endoRNases, the active sites of ToxN and Kid do not match well [11,25]. Less surprisingly, differences are also found in the regions that interact with their respective antitoxins. For instance, the helix 3 region of ToxN*_Pa_* overlays with the Kid structure but is greatly extended in the N-terminal section of the ToxN*_Pa_*, the main site of ToxI recognition. The equivalent helix in CptN*_Er_*, helix H4, is much shorter and, as such, resembles more closely to the Kid toxin (Figure 4A,D,E).

## 6. Functions of Type III TA System

Two functions have been ascribed to Type III TA systems so far. Historically, the first function was their abortive infection (Abi) activity that protects bacterial populations from invading bacteriophages. Their second known function involves their role in stabilisation of plasmids.

### 6.1. Abortive Infection

Some Type III systems are active protagonists in the “molecular arms race” that occurs between bacteria and phages. As these outnumber bacteria 10-fold in some environments, the prokaryotic hosts are potentially subject to constant viral predation [26]. In response to this acute selective pressure on microbial communities, bacteria have evolved a large array of defense mechanisms to circumvent the lethal impact of viral infections [27,28].

At least some of these Type III systems are bifunctional and can act as abortive infection (Abi) systems in addition to their TA functionality. This bifunctionality is possible as both activities require the involvement of a self-poisoning protein. Abi mechanisms rely on a toxic protein that can be viewed, from an evolutionary perspective, as inducing the altruistic suicide of infected cells to prevent phage propagation in bacterial populations. Even though 23 different Abi systems have been reported, mostly carried on lactococcal plasmids, there is still a paucity of information on the molecular mechanisms involved in their function, with the exception of TA/Abi systems for which some insight has been acquired recently [29]. Abi systems have been shown to act at different steps of the phage replication cycle i.e., from DNA replication to the lysis step—but ultimately they all lead to the death of the infected bacteria [29]. TA/Abi bifunctionality is not restricted to Type III systems as TA systems from Type I to IV have been shown to protect bacteria from bacteriophages [8,11,30,31,32]. While molecular details are still elusive in most cases, a working model is that—following toxin activation by the infecting phage—dissemination of phage progeny in the bacterial population is restricted. Toxin activation could rely on the differential stability of the antitoxin and toxin products. Post-infection, this characteristic of TA systems could favour a state of free toxins upon the depletion of the shorter lived antitoxins.

The ToxIN*_Pa_* system of *P. atrosepticum* was identified originally through its phage resistance capacity encoded by a cryptic plasmid. Subcloning of different plasmid fragments eventually narrowed the anti-phage activity down to the ToxIN system genetic module [11]. ToxIN*_Pa_* can inhibit a spectrum of phages infecting multiple Gram-negative enterobacteria such as *P. atrosepticum*, *Escherichia coli*, and *Serratia* spp. ([10] and unpublished data). This dual Abi and TA functionality was shown later to be shared by some other Type III TA systems. The chromosomal *tenpIN* locus from *Photorhabdus luminescens*, effectively aborts environmental coliphages when expressed from a plasmid in *E. coli* [11,18,33] and, likewise, AbiQ is effective, in Gram-positive *Lactococcus* *lactis*, against members of the common 936 and c2 phage groups as well as rarer lactococcal phages. When expressed in *E. coli*, AbiQ also protects this Gram-negative host from some coliphages [33]. Therefore, the protective effect against phages appears to be independent of the organism in which the systems are present [11,18,33]. Phage resistance may be also independent of the original genetic location as both plasmid and chromosomal systems can effectively inhibit phage replication [11,18]. Similarly, no correlation between the Type III TA systems families and Abi activity can be drawn currently. For instance, despite proficient anti-phage activity of the ToxIN*_Pa_* and AbiQ systems, no anti-phage activity could be shown for the closely related ToxIN*_Bt_* system in its native host [13] and while both phenotypes have been observed for members of the ToxIN family, no anti-phage activity has yet been observed for the two tested CptIN systems from *Eubacterium rectale* and *Ruminococcus torque* [14,18].

A further aspect of anti-phage activity of Type III systems is the specificity these systems show against subgroups of sensitive phages [11,18]. No correlation has been found between phage families (*Myoviridae*, *Siphoviridae*, and *Podoviridae*) and abortive infection [33,34]. It is not known whether phages that are not aborted by the TA/Abi systems have naturally and actively evolved resistance mechanisms against these systems in the perpetual arms race that occurs between them and their bacterial hosts, or whether they “simply” do not activate the toxin of these systems. Some examples of mechanisms selected by phages to avoid Abi systems in general (as well as TA/Abi systems) are known in the literature [34]. For instance, point mutations in key phage products have been identified as the basis of escape mechanisms. More recently it was shown that phage T4 has an ADP ribosyltransferase that chemically modifies the Type II MazF toxin to downregulate its activity and thus avoid this TA/Abi system [32]. Another phage escape mechanism includes two examples where the phages evolved mimics of the toxin’s substrates. In the case of the Type II RnlAB/LsoAB systems of *E. coli* and phage T4, the phage uses the Dmd protein which acts like a Type II antitoxin and directly interacts with the toxins. In contrast to the canonical antitoxins of RnlA and LsoA, Dmd is able to cross-neutralise several toxins. Recent crystallographic studies showed that Dmd is thought to have a different inhibition mechanism and directly interacts with the toxin active site by mimicking the toxin substrates [35].

In the Type III systems, the ToxIN-sensitive phage TE was shown to produce low frequency spontaneous mutants that escaped the ToxIN*_Pa_* system [36]. Analysis of the escape mutants revealed that the majority of the mutants had extended a short viral sequence similar to the repeats of the ToxI sRNA into a ‘pseudo-ToxI’ which functionally suppressed the toxin. In one case, recombination had allowed the phage escape mutant to obtain natural ToxI repeats from the original plasmid antitoxin sequences. Both scenarios allowed phage replication unaffected by the Abi/TA system [36].

The precise molecular mechanisms of these bifunctional TA/Abi systems are still under investigation. In agreement with the current model, the toxin endoRNase activity and the Abi phenotype have been shown to be linked [19]. However, this may not be a universal situation because mutagenesis of key toxin residues can lead to the loss of the Abi phenotype despite retained RNase activity [20] indicating that the details of the toxin activation are more subtle. This leads to the question as to how Type III TA/Abi systems are actually activated and the molecular basis of the differential sensitivity of different phages. Phage products that are involved in the activation of Type III TA/Abi systems might directly interact with the toxin, the antitoxin, or both, to prevent assembly of the TA heterocomplexes, or to disrupt them. Alternatively, a phage product might interrupt transcription of the TA operon or affect antitoxin RNA stability, thus causing imbalance in the TA components. Both scenarios would lead to an excess of free toxin, which could inhibit mRNA translation, leading to cell growth arrest and thereby ultimately prevent the release of phage progeny. The nature of the phage products involved and the way they interact (directly or indirectly) with the TA system is unknown. Experimental data for the ToxIN*_Pa_* and the AbiQ systems showed that toxin levels are not affected during phage infections nor is transcription of the TA systems increased [19,20]. However transcripts of the AbiQ system, expressed constitutively before infection, decrease during the infection, possibly as part of a general infection phenomenon [20]. Based on experimental data, it is thought that the TA systems are activated at late steps of phage replication, at least in the AbiQ system. It has been shown that phage DNA is replicated in infected cells in the presence of the AbiQ system, as indicated by the accumulation of concatemeric viral DNA [37].

### 6.2. Plasmid Inheritance through Addiction

Another function known for Type III systems is plasmid addiction. In contrast to the Abi phenomenon where TA systems protect bacteria from invading DNA, when acting as “addiction” modules they ensure the stable inheritance of plasmids in bacterial populations.

This feature of TA systems also relies on the antagonism of the self-poisoning essence of the toxin and the labile nature of the antitoxin [11]. It supposes a continuous synthesis of the antitoxin in order to keep the toxin in an inhibited state and thus renders the cell ‘addicted’ to the TA system. Consequently, when present on mobile genetic elements such as plasmids, these “addiction” modules promote the maintenance of the DNA molecule encoding the TA system and ensure the continued presence of plasmids in bacterial populations via killing of plasmid-free segregant cells by the toxins [15,38]. Historically, this mechanism has also been named post-segregational killing (PSK) as lethality arises when plasmids are not segregated into both daughter cells during cell division [38]. Alternatively, given the initially bacteriostatic—rather than bactericidal—nature of Type III toxins [11,20], mis-segregation could lead to a transitory growth inhibition. Bacteria that retained the plasmid would then be able to outgrow those that “lost” it, thus ensuring plasmid maintenance at the population level.

Plasmid addiction is a function found in many TA systems of Type I and II and has also been shown for some Type III TA systems. For example, ToxIN*_Pa_* and CptIN*_Er_* increased plasmid retention in *E. coli* W3110 to 100%, compared to 50% loss of the control vector [13,14]. Notably, the fact that CptIN*_Er_* is located on the chromosome of the original host, yet could promote plasmid maintenance when cloned, suggests that CptIN*_Er_* can promote its own retention and might be disseminated through horizontal gene transfer [14].

In addition to the post-segregational killing during the exponential growth phase, one Type III system has been show to ensure plasmid maintenance in stress conditions leading to sporulation of *Bacillus subtilis*. During sporulation, bacteria differentiate into spores, a metabolically dormant cell-type that can survive adverse environments and switch back to vegetative growth in favorable conditions. The frequency of plasmid loss during sporulation is higher than in vegetative growth [39] and the rate of plasmid loss can vary from 5% to 95% in *Bacillus* [40]. The *B. subtilis* ToxIN*_Bt_* system favours plasmid retention in this specific context by decreasing plasmid loss around 10-fold (from 58% for the control plasmid to 6% when the TA system was present) [39]. This was achieved by reducing the proportion of cells that form mature spores—probably by the action of the toxin in plasmid-free forespores. Only forespores that inherited a plasmid copy were able to mature and so this mechanism ensures plasmid maintenance throughout the environmental stress that precipitated sporulation. It is formally possible that other types of TA systems might affect plasmid retention by similar routes during sporulation [39].

## 7. New Bioinformatic Analysis Reveals a Significant Increase in Potential Novel Systems

Mining databases for ORFs and subsequent analysis of the proteins encoded by them can be a powerful tool to gain evolutionary insight into genes encoding TA systems [41,42]. Investigating the occurrence of Type III systems was initially complicated as bioinformatic analysis of non-coding RNAs is still problematic. Solving the structure of ToxN made it possible to identify new members of the Type III TA family by performing de novo structure-based homology searches [18]. A set of criteria was used to determine which ToxN structural homologues found by FUGUE [43], a program for sequence-structure comparison, are truly from Type III TA systems. The logic of the definition was as follows: the ToxN homologues should be preceded by a palindromic repeat acting as a Rho-independent terminator, which in turn should be preceded by sequences with a similar organization to *toxI*—composed of a tandem array of nucleotide repeats. Subsequently, samples from each family were taken and exhaustive BLASTp searches were performed, yielding a final list of 125 putative type III TA systems. The majority of hits were found on bacterial chromosomes and plasmids of Firmicutes, Fusobacteria, and Proteobacteria as well as Archaea. One *toxIN* locus was also encoded on a prophage [18]. Using these criteria, 37 putative Type III loci were identified and were divided into three independent families according to their protein sequences as described previously [18].

Given the near exponential increase of sequenced genomes available in databases during the last few years, we did a new BLASTp search. Using the amino acid sequences of the characterized ToxN*_Pa_*, ToxN*_Bt_*, AbiQ, TenpN*_Pl_*, and CptN*_Er_* toxins as input, we did an initial BLASTp search against the Bacteria and Viruses databases of Uniprot. We identified 603 potential Type III toxins with an *E*-value <0.001. A selection of these predicted toxins were further analysed following the previously established criteria. After validation as members of putative Type III TA systems, these toxins were used for a further round of BLASTp. The new round of BLASTp searches with the selected toxin homologues gave a few new putative toxins indicating that the search is exhaustive. The different toxin groups seem to cluster to particular phylogenetic groups. For instance, the ToxN family is primarily found in Firmicutes, Fusobacteria and occasionally other species. Systems from this group also seem associated with the mobilome as they can be found on plasmid and chromosomal locations, sometimes in the close vicinity of transposase elements. Despite the increased number of predicted systems, Type III TA systems appear largely restricted to a limited group of phyla as only 8 out of the currently estimated 74–76 bacterial phyla have at least one potential system [44]. Whether Type III TA systems are indeed only confined to a restricted number of bacteria, or whether the current distribution pattern is biased by the limited phylogenetic breadth of genome sequencing, is unknown. It is intriguing that Type III TA systems seem to be confined to a few phylogenetic groups. As the toxin mode of action, i.e., cleaving RNAs, is a general one that does not require any known external factors, and given that they are found on plasmids, one might expect them to be spread through more phylogenetic groups, in a similar way to the Type II systems where the majority of toxins are also RNases [41,42,45].

**Figure 5:**
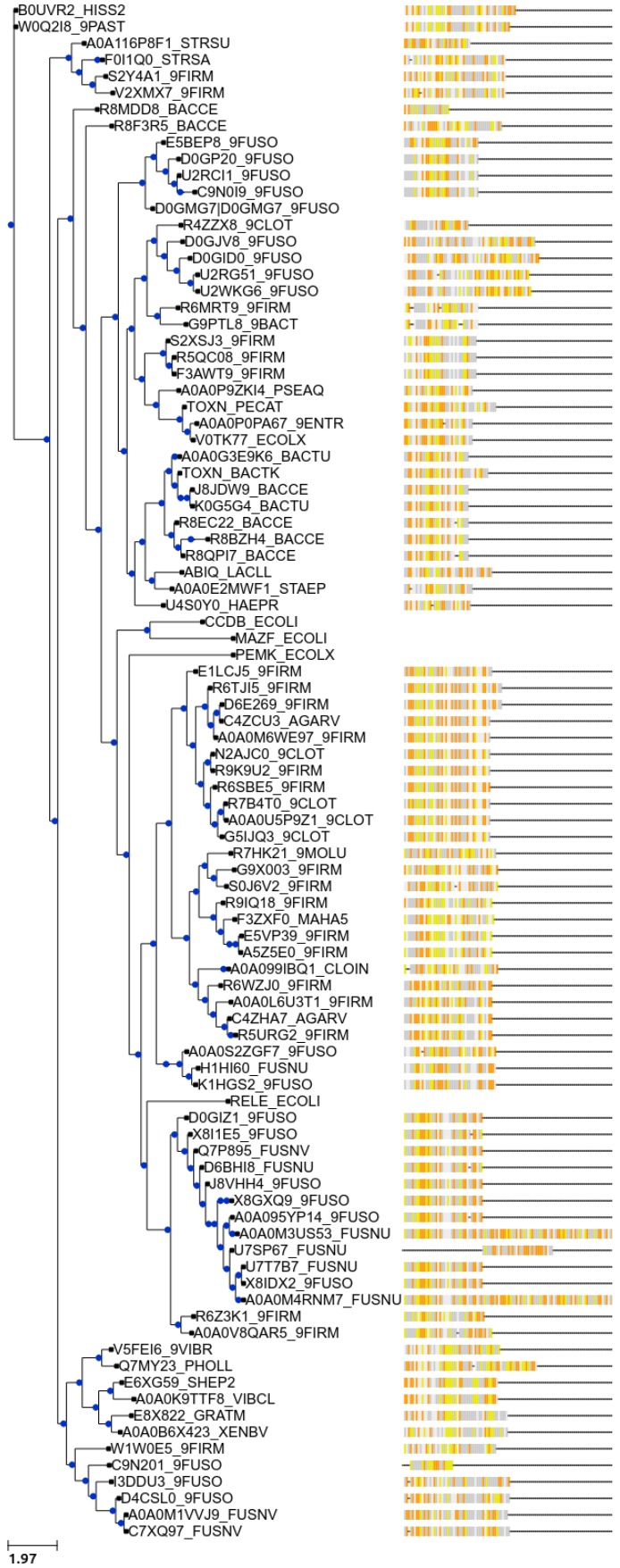
Phylogenetic tree based on representative toxins of putative Type III TA systems associated with the consensus repeats of the antitoxins: adenine (grey), guanine (white), cytosine (yellow), and uracil (orange). Toxin protein sequences were aligned using MUSCLE [48]. The tree has been constructed using PhyML and nearest neighbour interchange [49,50] and the figure has been made using Ete3 [51].

Advances in technologies such as single-cell genomics and metagenomics will enable the sequencing of uncultivated organisms from diverse habitats [44,46] and might affect the picture of TA system distribution as the current “tree of life” expands and culturing-associated biases are overcome [43,47]. When placing the consensus antitoxin repeats next to a phylogenetic tree based on the protein toxin sequences, it becomes clear that the antitoxins of closely related toxins are conserved as well (Figure 5). This consistency provided evidence for the notion of co-evolution of toxin and antitoxin components. The length and the primary sequence of the consensus repeats are usually quite conserved within the different toxin groups. On the other hand, the number of repeats is prone to slight variations.

## 8. Conclusions

Work in recent years has increased our understanding of bacterial Type III TA systems. However, it is abundantly clear that our understanding of the molecular mechanisms involved in Type III systems and their activation is still rudimentary. These TA systems provide valuable reagents for fundamental biochemical studies on protein:RNA interactions and the study of quaternary nucleoprotein complex assemblies. The ecological and fitness implications of bacterial carriage of Type III TA systems have yet to be investigated. Furthermore, the role of Type III systems in the evolution, replication, and physiology of both bacteria and their viral parasites warrants deeper consideration.
